# Supplementary material for: Quaternary Ammonium Groups Modified Magnetic Cyclodextrin Polymers for Highly Efficient Dye Removal and Sterilization in Water Purification
Source: Molecules. 2022 Dec 25;28(1):167. doi: 10.3390/molecules28010167 (PMC9822413; doi:10.3390/molecules28010167)
Supplement: Supplementary file 1 [file molecules-28-00167-s001.zip › molecules-2076374-supplementary.pdf]

Supplementary Materials

Quaternary Ammonium Groups Modified Magnetic Cyclodextrin Polymers for Highly Efficient Dye Removal and Sterilization in Water Purification

Bingjie Liu [1], Shuoxuan Wang [2], He Wang [2], Yong Wang [2,*], Yin Xiao [1,*] and Yue Cheng [2,*]

[1] School of Chemical Engineering and Technology, Tianjin University, Tianjin 300350, China
[2] School of Science, Tianjin University, Tianjin 300350, China
* Correspondence: wangyongtju@tju.edu.cn (Y.W.); xiaoyin@tju.edu.cn (Y.X.); chengyue90@tju.edu.cn (Y.C.)

Table S1. Detailed information of the pollutants used in the adsorption study.

Pollutants	Molecular structure	Molecular weight (g/mol)	Absorption peak (nm)
Methyl blue (MB)	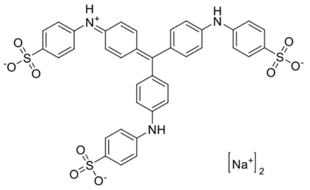	799.80	590
Orange G (OG)	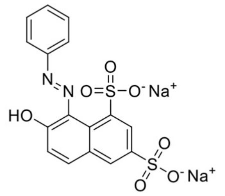	452.38	475
Bisphenol A (BPA)	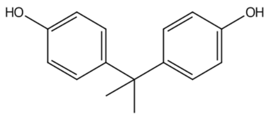	228.29	275

Table S2. Contents of iron in MCDP, QMCDP1, and QMCDP2.

Polymers	C_{Fe} (mg·mL^{-1})	ω_{Fe} (%)	ω_{MN} (%)
MCDP	0.148	29.6	40.8
QMCDP1	0.107	21.3	29.4
QMCDP2	0.079	15.8	21.8

Table S3. Maximum adsorption capacities for the adsorption of MB onto various adsorbents.

Adsorbents	Adsorption capacity ($mg\cdot g^{-1}$)	Reference
Graphene Aerogel	2.452	[1]
Tartaric acid modified wheat bran	25.18	[2]
Cyclodextrin–chitosan	50.12	[3]
Magnetic cyclodextrin–chitosan/graphene oxide	84.32	[3]
β-CD/PAA/GO	247.99	[4]
Graphene/β-CD composite	580.4	[5]
MCDP	125.88	This work
QMCDP1	278.57	This work
QMCDP2	174.50	This work

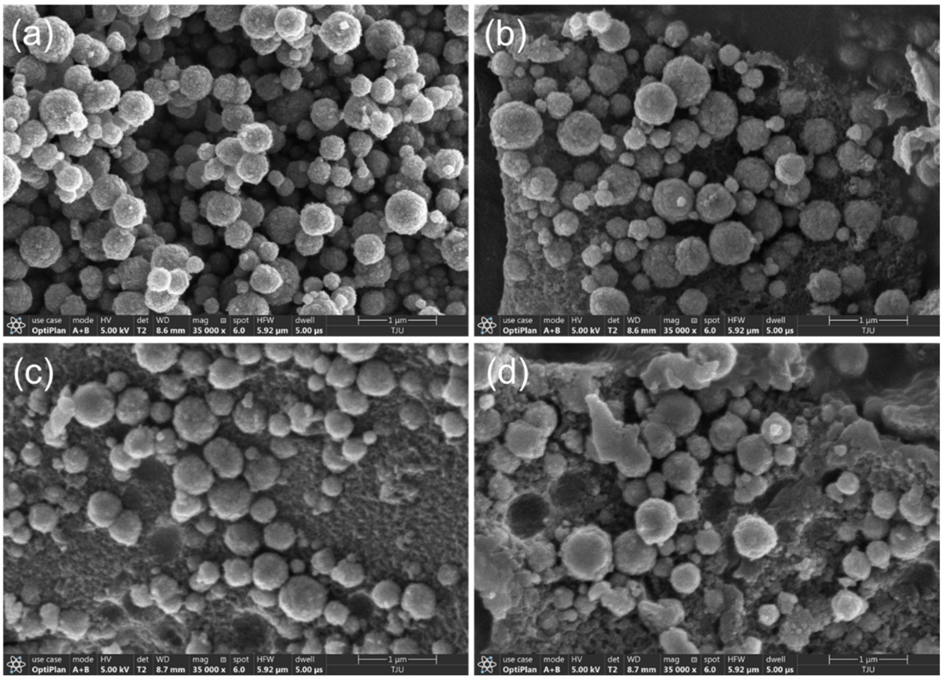

Figure S1. SEM images of **(a)** MN, **(b)** MCDP, **(c)** QMCDP1, and **(d)** QMCDP2

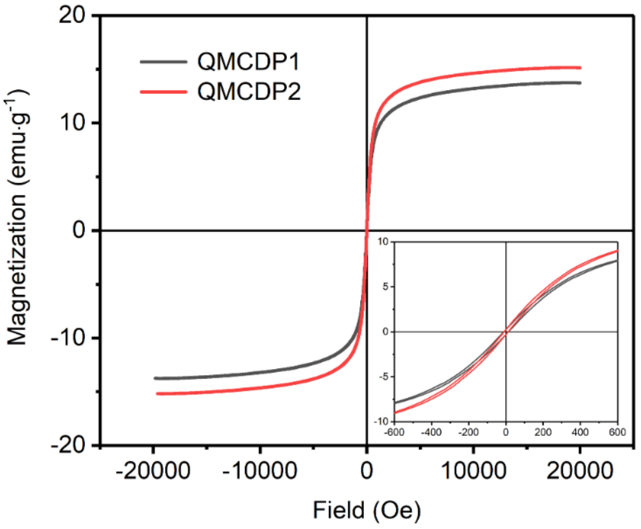

Figure S2. Magnetic hysteresis loops of QMCDP1 and QMCDP2 at 25 °C.

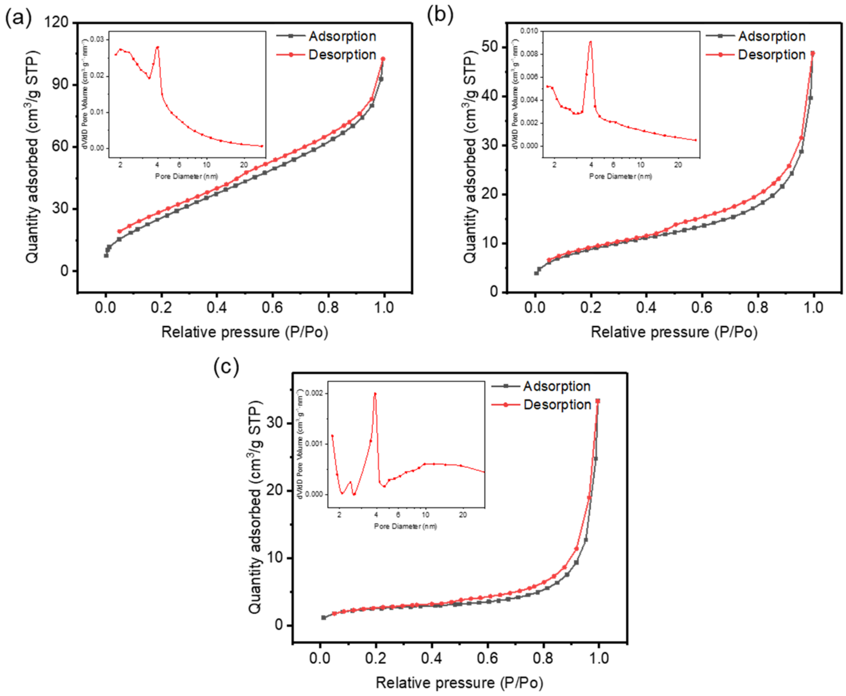

Figure S3. N_2 adsorption and desorption isotherms of **(a)** MCDP, **(b)** QMCDP1, and **(c)** QMCDP2, inset figures show the pore size distribution.

References

1. Song, X.; He, Y.; Pan, X.; Wei, M.; Li, R.; Zhou, X.; Zheng, Y.; Li, J.; Tang, H. Adsorptive Behavior of Methyl Blue on Graphene Aerogel: A Mechanism Study. *Journal Wuhan University of Technology, Materials Science Edition* **2021**, *36*, doi:10.1007/s11595-021-2400-3.
2. Yao, S.; Lai, H.; Shi, Z. Biosorption of Methyl Blue onto Tartaric Acid Modified Wheat Bran from Aqueous Solution. *Iranian J Environ Health Sci Eng* **2012**, *9*, doi:10.1186/1735-2746-9-16.

3. Fan, L.; Luo, C.; Sun, M.; Qiu, H.; Li, X. Synthesis of Magnetic β-Cyclodextrin-Chitosan/Graphene Oxide as Nanoadsorbent and Its Application in Dye Adsorption and Removal. *Colloids Surf B Biointerfaces* **2013**, *103*, 601–607, doi:10.1016/j.colsurfb.2012.11.023.
4. Liu, J.; Liu, G.; Liu, W. Preparation of Water-Soluble β-Cyclodextrin/Poly(Acrylic Acid)/Graphene Oxide Nanocomposites as New Adsorbents to Remove Cationic Dyes from Aqueous Solutions. *Chemical Engineering Journal* **2014**, *257*, doi:10.1016/j.cej.2014.07.021.
5. Tan, P.; Hu, Y. Improved Synthesis of Graphene/β-Cyclodextrin Composite for Highly Efficient Dye Adsorption and Removal. *J Mol Liq* **2017**, *242*, doi:10.1016/j.molliq.2017.07.010.
